# Lnc-PFAR and autophagy in chronic pancreatitis

**DOI:** 10.1080/27694127.2022.2047268

**Published:** 2022-03-22

**Authors:** Tao Zhang, Yu-Hang Sui, Guan-Qun Li, Bei Sun, Le Li

**Affiliations:** Department of Pancreatic and Biliary Surgery, The First Affiliated Hospital of Harbin Medical University, Harbin, China

**Keywords:** Autophagy, chronic pancreatitis, fibrosis, lnc-PFAR, *MIR141*, RB1CC1

## Abstract

Dysfunction of macroautophagy/autophagy has been demonstrated to contribute to multiple fibrotic diseases. In a recent study, we show that lnc-PFAR, a fibrotic-related lncRNA, is upregulated in human chronic pancreatitis tissues and mouse models and can serve as a biomarker for pancreatic fibrosis detection in the clinic. Indeed, our data reveal that lnc-PFAR affects autophagy activation through controlling *MIR141* maturation. Furthermore, lnc-PFAR binds with pre-*MIR141* and suppresses *MIR141* maturation, which releases RB1CC1 and induces autophagy activation. We address a novel perspective of lnc-PFAR-pre-*MIR141*-RB1CC1 axis in autophagy and pancreatic fibrosis, and discover a prospective pharmacogenomic biomarker for chronic pancreatitis. Our findings identify a potential therapeutic target in pancreatic fibrosis and provide more evidence to consider autophagy inhibitors for further application.

Pancreatic stellate cells (PSCs) are characterized as the major contributor in chronic pancreatitis (CP). Autophagy is required for the activation of PSCs through building stroma, accumulating extracellular matrix (ECM) and releasing cytokines. Our previous study has demonstrated that RB1CC1/FIP200/Atg17 expedites pancreatic fibrogenesis through autophagic activation. The RB1CC1-ULK1 complex promotes autophagosome formation and leads to excessive ECM accumulation, which eventually induces PSC activation and CP progression. Long non-coding RNAs (lncRNAs) have been revealed as key regulators of intrinsic processes; substantial attention has been gained from the competitive endogenous RNA (ceRNA) hypothesis, which postulates the conventional views of ceRNA mechanisms inducing dynamic changes in fibrotic diseases. Although fibrosis-related lncRNAs have been identified in CP, the mechanisms that cause and maintain sustained PSC activation remain unclear.

In our recent study, a pro-fibrotic lncRNA (NONMMUT096607.1), named lnc-PFAR, was identified in activated PSCs by transcriptome sequencing [1]. Our data indicate that lnc-PFAR affects autophagy and pancreatic fibrosis i*n vitro and in vivo*. Lnc-PFAR can only coordinate with the effect driven by TGFB in PSCs activation, which revealed that it acts as a fibrotic cooperator. LncRNAs share miRNA response elements and approach the posttranscriptional regulation of protein-coding genes. The differential miRNAs between normal pancreas and CP tissues were compared to predict the downstream cascades and give pro-fibrotic insights. We proposed a mechanism that lnc-PFAR inhibits *MIR141* maturation and then accelerates the consequences on PSC activation. Our study demonstrated that lnc-PFAR binds with pre-*MIR141* and suppresses the mutation of *MIR141*, which releases RB1CC1 in the cytoplasm and facilitates a pro-autophagic process. In the CP mouse model, blockade of lnc-PFAR shows encouraging effects on delaying pancreatic fibrosis, and dramatically alleviating systemic inflammatory response. Meanwhile, autophagosome formation is decreased and the phosphorylation of ULK1 is increased, consist with our previous publication.

The clinical samples were then used to figure out the roles of lnc-PFAR, pre-*MIR141* and *MIR141* on evaluating pancreatic fibrosis, and to investigate the potentially non-invasive biomarker for early detection of pancreatic fibrosis. We observed colocalization among lnc-PFAR, pre-*MIR141* and *MIR141* in CP tissues by fluorescence in situ hybridization. In addition, lnc-PFAR is found increasingly expressed in CP samples and plasma of CP patients, and can serve as a biomarker for fibrotic evaluation. In sum, lnc-PFAR restrains the mutation of pre-*MIR141* and increases the cytoplasmic RB1CC1 level, suggesting novel regulatory roles of the lnc-PFAR-pre-*MIR141*-RB1CC1 axis in PSC autophagic activation and CP progression.

Targeting autophagy has been reported to be a promising strategy for alleviating fibrotic diseases. However, several points should be further discussed. First, PSCs have been recognized as the main cell type for CP initiation, most studies aim to illustrate the potential roles of autophagy in PSC activation, and a few studies tried to link the regulatory effects of autophagy between acinar cells and pancreatic fibrosis. We speculate that understanding the autophagy-induced crosstalk or cell communications between acinar cells and PSCs may become hot topics.

Second, CP elicits immune activation or deficiency, which involves both the innate and adaptive immune response. As a result, increased mononuclear cells (particularly T cells, neutrophils and macrophages) are infiltrated in the pancreatic tissues, and large amounts of cytokines are secreted. Investigating the blockade of autophagy on immune cell infiltration, activation, and even exhaustion will provide new thoughts about autophagy activation and the adaptive immune response (Figure 1).

**Figure 1.**
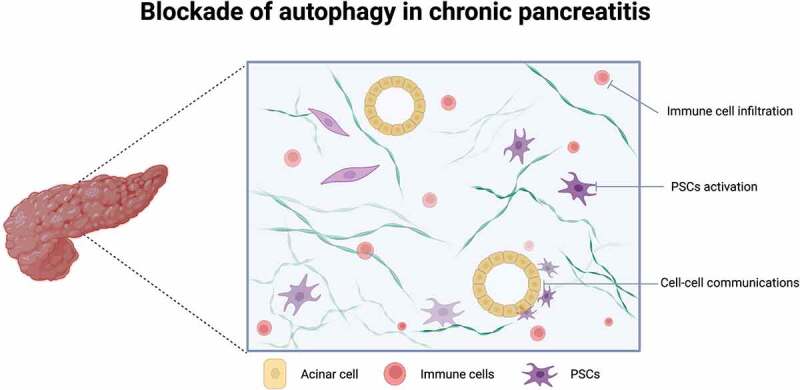
Three potential targets of autophagic inhibition to alleviate pancreatic fibrosis, including pancreatic stellate cell (PSC) activation, immune cell (macrophages, neutrophils) infiltration, and cell communication between acinar cells and other subtypes (PSCs, immune cells or ductal cells).

Third, the histological diagnostic method is commonly used for pancreatic fibrosis evaluation. Our study suggests that lnc-PFAR is highly increased in CP patients’ plasma, which suggests that testing the plasma lnc-PFAR expression could be a non-invasive way for evaluating pancreatic fibrosis. Furthermore, the sample size should be enlarged and the specificity and sensitivity of lnc-PFAR need to be verified in precise diagnosis, which may contribute to discovering a pharmacogenomic biomarker.

Fourth, several clinical trials have revealed striking results of ameliorating fibrotic diseases by autophagy inhibitors in idiopathic pulmonary fibrosis, hepatic cirrhosis and kidney fibrosis. None of the FDA-approved autophagy inhibitors, including hydroxychloroquine, rapamycin and nimbolide, have been explored to prevent, retard, or reverse pancreatic fibrosis. More evidence needs to be obtained to support further application in patients. Taken together, the framework establishes the role of lnc-PFAR-induced autophagy on pancreatic fibrosis and approaches the diagnosis and therapeutic targets for alleviating CP progression.
